# Intraocular Pressure rise after Anti-VEGF Treatment: Prevalence, Possible Mechanisms and Correlations

**DOI:** 10.5005/jp-journals-10008-1132

**Published:** 2013-01-15

**Authors:** George Kampougeris, Dimitrios Spyropoulos, Adrianna Mitropoulou

**Affiliations:** Private Practice, Department of Ophthalmology, Greece; Department of Ophthalmology, Athens Medical Center, Greece; Department of Ophthalmology, Athens Medical Center, Greece

**Keywords:** Anti-VEGF injections, Neovascular AMD, Intraocular pressure rise.

## Abstract

Intraocular pressure (IOP) rise after anti-vascular endothelial growth factor (VEGF) treatment for neovascular age-related macular degeneration (AMD) can be either short-term or long-term and may require medical intervention. Short-term IOP spikes are a fairly common and well recognized complication of anti-VEGF injections. Long-term IOP rise is less well-understood and disputed as a complication by some authors. We try to review current literature on the subject and especially studies focused on the prevalence of this complication, speculate on possible mechanisms of IOP rise and discuss correlations of long-term IOP rise with the nature of the injected agent, average number of injections, previous glaucoma history and other factors.

**How to cite this article:** Kampougeris G, Spyropoulos D, Mitropoulou A. Intraocular Pressure rise after Anti-VEGF Treatment: Prevalence, Possible Mechanisms and Correlations. J Current Glau Prac 2013;7(1):19-24.

## INTRODUCTION

Intravitreal antivascular endothelial growth factors (anti-VEGFs) are currently the mainstay of treatment for choroidal neovascularization due to age-related macular degeneration (AMD). Based on well-conducted studies,^1-3^ ranibizumab (Lucentis, Genentech, San Francisco, CA, USA) and bevacizumab (Avastin, Genentech) are used all over the world on many thousands of patients with this debilitating disease. Ranibizumab is a F_ab_ fragment of a recombinant humanized immunoglobulin, targeting VEGF-A and has Food and Drug Administration (FDA) approval for treating wet AMD. Bevacizumab is a full size humanized monoclonal antibody targeting VEGF-A and is approved by the FDA for the treatment of various cancers, though not specifically for intravitreal use. The widespread use of both these agents in the eye is not without side effects, however, ranging from innocuous subconjunctival hemorrhage to devastating endophthalmitis. One of these side effects has come into focus lately and is related with the rise of intraocular pressure (IOP), which can be transient or permanent. We will try to address this topic providing insight to the prevalence of IOP rise related to anti-VEGF use based on reports on such cases so far, possible mechanisms implicated and proposed prevention strategies.

## SHORT-TERM IOP INCREASE

A transient increase in IOP is a well-known complication of intravitreal injections (whether they are anti-VEGF or not) and has been reported in the initial MARINA and ANCHOR studies^1,2^ for ranibizumab treatment, although in only a few patients. This is probably related to the immediate increase of intraocular volume together with factors, such as scleral rigidity and is corroborated by the fact that hyperopic (shorter and more rigid) eyes show a greater rise in IOP immediately postinjection than longer eyes.^4^ Other studies have confirmed these findings^5-8^ and have actually reported that an immediate increase in IOP after anti-VEGF injections is extremely common. Gismondi et al^4^ reported that 88.9% of eyes (54 eyes in total) had an IOP of more than 30 mm Hg 5 seconds postinjection, whereas Kim et al^5^ reported a mean IOP immediately postinjection of 44 mm Hg (120 eyes in total) with 36% of eyes exceeding 50 mm Hg. All relevant studies taken together, it is clear that the time of IOP measurement postinjection is strongly related with IOP rise, since the greatest IOP values are measured immediately after the injection, with IOP values coming down with longer intervals between injection and IOP measurement. It is reassuring however that in all these studies, IOP returned to normal or near-normal levels 30 to 60 minutes postinjection. Of note is that eyes with a known history of glaucoma were at greater risk of having longer time to recover their preinjection IOP levels,^5^ possibly because these eyes had already reduced outflow facility. Since, treatment with anti-VEGF injections cannot be withheld from eyes with wet AMD, various strategies have been proposed to avoid these short-lived high IOPs. One is pretreatment with brimonidine 0.2%/timolol 0.5% drops^9^ (mean postinjection IOP was 28.4 mm Hg in treated eyes compared to 34.1 mm Hg in placebo eyes, 5 minutes postinjection). Another is the use of ocular decompression with a mercury bag^10^ (mean IOP reduced from 44.5 to 20.6 mm Hg in patients receiving intravitreal ganciclovir injections). Although transient IOP spikes are not putting visual function at risk in otherwise healthy eyes,^11^ there have been reports of visual field progression after such spikes postcataract surgery.^12^ We suggest that glaucomatous eyes (higher risk eyes) should be identified before anti-VEGF injections and monitored carefully postinjection for IOP spikes that can cause visual field deterioration. We use ocular digital massage for half to 1 minute before anti-VEGF injection in all eyes. This simple measure has blunted excessive IOP spikes in our experience (anecdotal data). It is ideal (though not always feasible in a busy practice) to measure IOP 15 to 30 minutes postinjection in all eyes and treat high IOP accordingly, even with anterior chamber paracentesis.^13^

## LONG-TERM IOP INCREASE

Long-term or sustained IOP rise after anti-VEGF injections appears to be a more complex problem. Definition of this term is somehow varied in relevant studies but most of them agree on the following: A previously normotensive eye undergoing anti-VEGF injections should exhibit IOP measurements above 21 mm Hg on two consecutive visits or an increase of 5 mm Hg in IOP from baseline preinjection measurements. Some authors include eyes showing IOP measurements >25 mm Hg at some point postinjection, requiring continuous IOP lowering treatment. IOP was generally measured at every office appointment (before any new anti-VEGF injection, if deemed necessary), so these measurements were at least a month apart from any previous injection.

## PREVALENCE OF LONG-TERM IOP INCREASE

The prevalence of long-term IOP rise after anti-VEGF injections is reported as variable in the literature. The original MARINA and ANCHOR trials^1,2^ did not find any significant long-term effects on IOP after 2 years of anti-VEGF treatment. On the other hand, a post hoc analysis showed that 2.1% of eyes in the MARINA and 3.6% of the eyes in the ANCHOR trials met the criteria for long-term IOP rise at the 2 years follow-up, compared with 0.4% and 0% of eyes in the control groups respectively.^14^ Bakri et al^15^ first reported sustained IOP rise after ranibizumab injections requiring medical treatment in a small series of four patients and Kahook et al reported similar findings in six patients.^16^ Several studies followed reporting prevalence of long-term IOP rise after treatment with ranibizumab and/ or bevacizumab injections.^17-22^

Adelman et al^17^ reported a prevalence of four of 116 in patients receiving anti-VEGF injections (3.45%). In their study, among the 116 patients with wet AMD, who received intravitreal injections, 57 received ranibizumab only, 40 received bevacizumab only and 19 received both agents. None of the four patients had a history of glaucoma or ocular hypertension (OHT). The mean number of anti-VEGF injections prior to raised IOP was 13.3 (range, 3-19). Good et al^18^ reviewed 215 eyes receiving anti-VEGF treatment and reported 13 eyes (6%) in total requiring IOP lowering intervention. In eyes receiving ranibizumab only the prevalence was 3.1% (3/96 eyes) and in eyes receiving bevacizumab only the prevalence was 9.9% (10/101 eyes), the difference between the two drugs barely significant (p = 0.049). In this study, it was found that patients with pre-existing glaucoma experienced higher rates of elevated IOP compared with patients without pre-existing glaucoma (33 *vs* 3.1% respectively, p < 0.001). Of note also, that the glaucoma subgroup had a lower median number of injections compared with the nonglaucoma group (6 and 9.5 respectively, p = 0.031). Hoang et al^19,20^ presented data from a cohort of patients, the second paper with additional data on more patients. In their first study, the rate of long-term raised IOP after anti-VEGF injections was reported in uniocularly-injected patients (the other eye served as control). According to the results, 11.6% of treated *vs* 5.3% of untreated/control eyes experienced long-term IOP elevation (p = 0.02). Of the factors considered, only the total number of injections showed a statistically significant association with IOP elevation (p = 0.05). In the second paper, they presented data on 449 eyes (328 patients) receiving anti-VEGF injections, this time including patients injected bilaterally. Again they reported association with the total number of injections, with increased odds ratio (OR = 16.1, p = 0.008) of sustained IOP elevation in eyes receiving ≥ 29 injections compared with eyes receiving ≤ 12 injections. The authors report that the study was not adequately powered to distinguish differences between patients receiving ranibizumab *vs* bevacizumab.

Wehrli et al^21^ on the contrary reported a low prevalence of long-term IOP raise after anti-VEGF injections. In their study, they included data from 302 injected eyes and 226 control (noninjected), fellow eyes. Of the 302 eyes with wet AMD, 270 were nonglaucomatous and 32 had glaucoma. These groups received on average 8.4 and 7.9 anti-VEGF injections respectively, whereas 34% of the eyes were treated exclusively with ranibizumab, 32% received bevacizumab only and 34% received both agents. Overall, 5 of 302 injected eyes developed long-term IOP increase compared with 7 of 226 control eyes, with a median time to development of peak IOP after the last injection of 14 weeks. The median number of injections before peak IOP was 8. None of the 26 patients in this study, who received more than 20 injections developed the aforementioned complication. They concluded that the incidence of developing sustained IOP increase per eye-year was 0.51% for injected eyes compared to 1.00% in control eyes, a difference that was not statistically significant [hazard ratio = 0.48; 95% confidence interval (CI): 0.11-2.23]. In eyes with concomitant glaucoma, the incidence was 3.1% per eye-year compared to 5.7% in nonglaucomatous eyes (difference: Not statistically significant, hazard ratio = 0.59; 95% CI: 0.10-3.60).

**Table 1:** **Table 1:** An overview of the findings of relevant studies in the literature. Complicated eyes are defined as eyes developing long-term IOP increase after anti-VEGF injections and uncomplicated eyes as eyes without long-term IOP increase after treatment

Overview of findings in studies reporting long-term IOP increase afteranti-VEGF administration																							
*Study*		*Controls*		*Prevalence*								*History of glaucoma or OHT*							*Average number of injections at time of complication*					
				*All eyes*		*Ranibizumab only eyes*		*Bevacizumab only eyes*				*Both agents eyes*		*Complicated eyes*		*Uncomplicated eyes*			*IOP range (complicated eyes)*		*Complicated eyes*		*Uncomplicated eyes*
Adelman et al^17^		No		3.45% (4/116)		1.75% (1/57)		2.5% (1/40)				10.5% (2/19)		0%					28-36 mm Hg		13.3		
Goodetal^18^				6% (13/215)		3.1% (3/96)		9.9% (10/101)				0%(0/18)		33% (7/21)		3% (6/194)			23-36 mm Hg		5		9.5
Hoang et al^20^		Yes (fellow eyes)		7.1% (32/449)																	25.8		20.8
Wehrli et al^21^		Yes (fellow eyes)		1.6% (5/302)		0.96% (1/104)		1.05% (1/95)				2.9% (3/103)		6.25% (2/32)		1.1% (3/270)			25-29 mm Hg (1 eye with 60 mm Hg)		8		
Mathalone et al^22^		No		11 % (22/201)				11% (22/201)						6.3% (1/15)		11.3% (21/186)			22-36 mm Hg		5		4

Mathalone et al^22^ reported the prevalence of sustained IOP elevation on 201 eyes (174 patients) treated exclusively with bevacizumab for wet AMD and they found it to be 11% (22 eyes). After multivariable regression analysis, they reported that male gender [odds ratio (OR) = 3.1, 95% CI (1.1-8.5), p = 0.029] and length of interval between injections [OR = 3.0, 95% CI: 1.1-7.9, p = 0.028) were risk factors for eyes having raised IOP. The prevalence of IOP elevation was significantly higher when the interval between injections was less than 8 weeks compared to more than 8 weeks (17.6 and 6% respectively, p = 0.009). They did not find any association with pre-existing glaucoma (p = 0.9). In this study, the number of injections prior to IOP elevation in affected eyes was not significantly different from that for eyes without sustained IOP elevation (median: 5, range: 3-18 and median: 4, range: 3-22 respectively, p = 0.34).

Tseng et al^23^ reported mainly on characteristics and treatment of eyes with long-term IOP increase after anti-VEGF treatment. One of the doctors participating in this study treated most eyes and prevalence of this complication is reported in his series as 3.4% (19/555 patients).

All the above studies share a common point and that is their retrospective nature with all inherent restrictions present in retrospective studies. They provide though a useful insight in cases with long-term IOP increase after anti-VEGF injections and their management. Together with studies^23,24^ which focus specifically on treatment of these cases, they provide evidence that most eyes are well controlled after medical or laser treatment (SLT) with a few exceptions^24^ requiring trabeculectomy. An overview of the studies reporting prevalence and possible causative factors of long-term IOP increase after anti-VEGF treatment is presented in Table 1.

## POSSIBLE MECHANISMS FOR LONG-TERM IOP INCREASE

The mechanism of long-term IOP rise associated with anti-VEGF treatment is more difficult to ascertain than the one of short-term IOP spikes and is possibly multifactorial. There have been conflicting results in studies investigating direct toxicity of these agents^25,26^ in cells and structures of the eye. A case report^27^ suggested possible inflammation in the trabecular area as a direct cause of increased IOP, but this was not observed in eyes from larger series.

There has also been some speculation regarding differences in the prevalence (and the mechanism implicated) between the two anti-VEGF agents used nowadays, i.e. between ranibizumab and bevacizumab. Bevacizumab has a molecular weight of 149 kDa and is three times larger than ranibizumab (48 kDa). It has been suggested^15^ that these molecules (bevacizumab in particular, both because of size and longer half-life in the vitreous) can accumulate in the trabeculum and/or Schlemm's canal, especially after long-term repeated administration and cause either direct obstruction or indirect change of outflow facility. Another mechanism that has been proposed involves possible protein aggregates and/or silicone droplets contamination and outflow obstruction from the syringes used. It is possible that this contamination increases by freezing/thawing of anti-VEGF agents or any other mishandling during storage.^28-30^ In addition, ranibizumab comes in a single dose vial ready for use which is drown into the syringe immediately before injection, while bevacizumab is drown from a larger vial, usually in multiple syringes, which are frozen for variable time periods, therefore increasing the risk not only of aggregates but of sterile and infectious endophthalmitis as well. In a recent study,^31^ it was noted that patients receiving bevacizumab were 12 times more likely to develop severe intraocular inflammation following each injection than those who received ranibizumab. In many countries, this procedure is carried in compounding pharmacies and, therefore, ophthalmologists do not interfere with drug storage and preparation but just deliver the injection. In the Good et al study,^18^ there was a disparity in long-term IOP increase in eyes injected with bevacizumab between the two centers participating in the study. The authors attributed these findings to the fact that two different compounding pharmacies were bevacizumab providers for the two centers, with differences in preparation, freezing time and storage procedures. A similar explanation is noted in the Mathalone et al paper,^22^ although in their study storage time did not exceed 3 days. In the Wehrli et al paper,^21^ despite storage time for bevacizumab being up to 2 weeks, no significant long-term IOP increase was noted.

Total number of received injections has also been implicated as a causative factor. Good et al did not find any association. Tseng et al^23^ report a series of eyes that developed glaucoma after a mean of 20 injections (range: 8-40), whereas in the other studies, the number of eyes with more than 20 injections is low. Hoang et al^20^ on the contrary, reported the total number of injections as being a risk factor, especially if the number of injections exceeded 29 (16.1 times the risk than eyes receiving fewer than 12 injections). Of importance here, is the large number of eyes that received more than 22 injections (224 eyes), whereas in other studies, the number of eyes with more than 22 injections is much lower.

The interval between injections has also been reported to be important for long-term IOP raise. Mathalone et al found that, if the interval was less than 8 weeks, the risk was higher. Good et al did not observe any correlation. Two of the eyes in the Tseng paper returned to normal IOPs after switching from ‘regular interval' dosing to a kind of ‘treat and extend' dosing without any other treatment, suggesting that increasing the interval between injections may allow the agent to be cleared from the eye and thus IOP normalization.

Previous glaucoma or OHT has been implicated as a causative factor for postinjection long-term IOP raise. Good et al noted this finding and suggested that eyes with an already compromised aqueous humor outflow system may be more prone to developing elevated IOP in this setting. Wehrli et al report no association and Adelman et al report no previous glaucoma or OHT in any affected eye in their series. Mathalone et al did not report any association but raise the posibility of having a small number of glaucomatous eyes in their study. Hoang et al also did not report any association but note that inclusion criteria in their study excluded three patients with pre-existing glaucoma with poorly controlled IOP at baseline.

## CONCLUSION

It is difficult to have definite conclusions because of the many variables implicated, lack of controls in most and retrospective nature of all available studies, sometimes excluding any relation between injections and long-term increased IOP. Different anti-VEGF agents, combination of the two agents, variable schemes of treatment with differences in intervals between injections and their total number, differences in preparation, handling, freezing and storage for bevacizumab, possible implication of the syringes used (silicone free or not), possible relation with previous glaucoma or OHT, are all factors that may come into play for eyes developing IOP increase. Currently, physicians undertaking treatment with anti-VEGF agents should be vigilant for this complication and monitor IOP in every visit (before any treatment), especially in eyes with relevant history. In cases of treatment with bevacizumab, there must be caution when the injection is stored for a long time. Another suggestion is to treat, when it is possible, in a ‘treat and extend' regimen, extending in this way the interval between injections and possibly reducing the total number of injections. Prospective studies with long follow-up will be needed to establish the true prevalence and possible correlations of this complication.
